# The Transient Receptor Potential Ion Channel TRPV6 Is Expressed at Low Levels in Osteoblasts and Has Little Role in Osteoblast Calcium Uptake

**DOI:** 10.1371/journal.pone.0028166

**Published:** 2011-11-29

**Authors:** Robert Little, Richmond Muimo, Louise Robson, Kate Harris, Peter S. Grabowski

**Affiliations:** 1 Department of Human Metabolism, The Mellanby Centre for Bone Research, The University of Sheffield, Sheffield, United Kingdom; 2 Department of Infection and Immunity, The University of Sheffield, Sheffield, United Kingdom; 3 Department of Biomedical Science, The University of Sheffield, Sheffield, United Kingdom; Indiana University School of Medicine, United States of America

## Abstract

**Background:**

TRPV6 ion channels are key mediators of regulated transepithelial absorption of Ca^2+^ within the small intestine. *Trpv6* ^-/-^ mice were reported to have lower bone density than wild-type littermates and significant disturbances in calcium homeostasis that suggested a role for TRPV6 in osteoblasts during bone formation and mineralization. TRPV6 and molecules related to transepithelial Ca^2+^ transport have been reported to be expressed at high levels in human and mouse osteoblasts.

**Results:**

Transmembrane ion currents in whole cell patch clamped SaOS-2 osteoblasts did not show sensitivity to ruthenium red, an inhibitor of TRPV5/6 ion channels, and ^45^Ca uptake was not significantly affected by ruthenium red in either SaOS-2 (*P* = 0.77) or TE-85 (*P* = 0.69) osteoblastic cells. In contrast, ion currents and ^45^Ca uptake were both significantly affected in a human bronchial epithelial cell line known to express TRPV6. TRPV6 was expressed at lower levels in osteoblastic cells than has been reported in some literature. In SaOS-2 TRPV6 mRNA was below the assay detection limit; in TE-85 TRPV6 mRNA was detected at 6.90±1.9 × 10^−5^ relative to B2M. In contrast, TRPV6 was detected at 7.7±3.0 × 10^−2^ and 2.38±0.28 × 10^−4^ the level of B2M in human carcinoma-derived cell lines LNCaP and CaCO-2 respectively. In murine primary calvarial osteoblasts TRPV6 was detected at 3.80±0.24 × 10^−5^ relative to GAPDH, in contrast with 4.3±1.5 × 10^−2^ relative to GAPDH in murine duodenum. By immunohistochemistry, TRPV6 was expressed mainly in myleocytic cells of the murine bone marrow and was observed only at low levels in murine osteoblasts, osteocytes or growth plate cartilage.

**Conclusions:**

TRPV6 is expressed only at low levels in osteoblasts and plays little functional role in osteoblastic calcium uptake.

## Introduction

Skeletal mineral includes a dynamic store of calcium ions that can be accessed rapidly in the absence of dietary sources to maintain the plasma concentration within tightly regulated limits. Calcium homeostasis involves both active and passive absorptive mechanisms in the intestine, both constitutive and regulated reabsorptive processes in the kidney and the exchange between mineralized and ionized forms of calcium in bones [1], [2]. Two of the key molecules that mediate intestinal absorption and renal reabsorption of Ca^2+^ have recently been identified. TRPV6, a Ca^2+^ selective member of the transient receptor potential vanilloid class of ion channels, was detected in epithelial cells of the small intestine and was described as “the gatekeeper” of active Ca^2+^ absorption from the diet [3], [4], [5], [6]. TRPV5, a closely related homologue, was identified as the principal mediator of regulated Ca^2+^ reabsorption in the distal convoluted tubules of the kidney [7]. Ca^2+^ is transferred from the intestinal or the renal tubular lumen into epithelial cells via these key ion channels which are expressed on the apical cell surface, transported through the cell complexed with the Ca^2+^ binding proteins calbindin–D_9K_ and –D_28K_, and is extruded through the basolateral membrane via the Ca^2+^ exchangers NCX1 and PMCA1b [8], [9], [10].

Mice in which *Trpv6* is ablated globally (*Trpv6* ^-/-^) were reported to exhibit 60% lower intestinal Ca^2+^ absorption, secondary hyperparathyroidism, a greater than 2-fold higher Ca^2+^ excretion and 9.3% lower femoral bone mineral density (BMD) compared with wild type (WT) littermates [11]. The low BMD in these mice is consistent with the proposed role of TRPV6 in intestinal and renal absorption of Ca^2+^ as a consequence of reduced Ca^2+^ availability for mineralization. However, in their study, Bianco *et al*. [11] were unable to rescue the BMD of *Trpv6* ^-/-^ mice with a high (2%) Ca^2+^ diet and the relative increase in BMD in *Trpv6* ^-/-^ mice on this diet was lower than in wild-type littermates [11]. On a low Ca^2+^ diet, *Trpv6* ^-/-^ mice did not increase their urinary deoxypirodinoline excretion. The authors argued these findings showed that the bones and kidneys of *Trpv6* ^-/-^ mice do not respond to parathyroid hormone (PTH) and 1,25-dihydroxy vitamin D3 (1,25D3), implying a role for TRPV6 in both tissues.

A number of previous studies report that osteoblasts express all the key molecular components of the “transepithelial” calcium transport pathway. Balmain *et al.* [12] provided the first immunohistological evidence that calbindin–D_9K_ is expressed by both osteoblasts and osteocytes and that its synthesis in trabecular bone is regulated by 1,25D3. Berdal *et al*. [13] showed that both calbindin–D_9K_ and –D_28K_ are expressed in rat osteoblasts, while Faucheux *et al*. [14] demonstrated the production of calbindin-D_28K_ during mineralization in human bone marrow stromal cells. Lundquist *et al*. [15] demonstrated the expression of three isoforms of the NCX1 Na^+^/Ca^2+^ exchanger in primary rat odontoblasts and osteoblasts and in a rat and a human osteoblastic cell line. Meszaros and Karin [16] showed that primary rat osteoblasts and osteoblast-like osteosarcoma cells express PMCA1b, an isoform of the plasma membrane calcium ATPase. Weber *et al*. [17] demonstrated that ECaC2 (TRPV6) and calbindin-D9K, but not ECaC1 (TRPV5) or calbindin-D28K, were strongly expressed in primary murine calvarial osteoblasts and whole bones. Nijenhuis *et al.* [18] also detected mRNA for TRPV6 in whole murine bone. Taken together, the evidence suggested that a complete mechanism existed for the involvement of TRPV6 in osteoblastic calcium handling, and led us to hypothesize that transmembrane Ca^2+^ ion currents and Ca^2+^ uptake in osteoblastic cells would be sensitive to ruthenium red, an inhibitor of both TRPV6 and TRPV5 [19]. We attempted to characterize the contribution of TRPV6 to Ca^2+^ membrane currents in whole cell patch clamped SaOS-2 human osteoblastic cells but we failed to demonstrate ruthenium red sensitivity. We also did not observe an effect of ruthenium red on ^45^Ca uptake in either SaOS-2 or TE-85 osteoblastic cells. On further investigation we found little or no expression of TRPV6 in a variety of human and murine osteoblastic cells using both qPCR and immunohistochemistry. We show here that TRPV6 is not widely expressed in osteoblastic cells and that it plays little role in osteoblastic Ca^2+^ uptake. Our functional data are fully consistent with a recent report that TRPV6 plays little role in bone mineralization [20], but our TRPV6 expression data contrast strongly with that of Weber *et al*. [17] and van der Eerden *et al*. [20].

## Results

### Electrophysiological characterization of membrane currents

**Figure 1:**
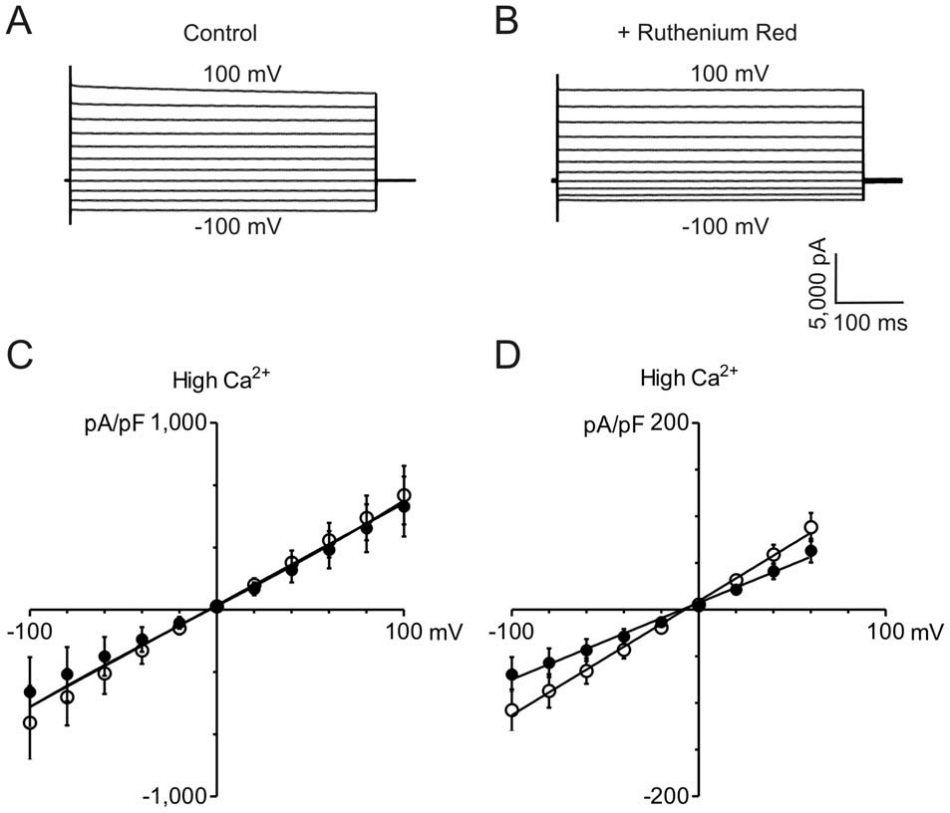
Currents in whole cell voltage-clamped SaOS-2 and 16HBE14o- cells. A, B: typical traces obtained from the same SaOS-2 cell in 2 mM [Ca^2+^]_O_ without (A) and with (B) 100 µM ruthenium red. C: Currents for SaOS-2 cells (mean ± SEM; n = 5) in 2 mM [Ca^2+^]_O_ without (open symbols) and with (solid symbols) 100 µM ruthenium red. A straight line adequately fits both conditions (*P* = 0.21; comparison of both datasets using the extra sum of squares F-test). D: Currents for 16HBE14o- cells (mean ± SEM; n = 15) in 2 mM [Ca^2+^]_O_ without (open symbols) and with (solid symbols) 100 µM ruthenium red. (*P* = 0.027; extra sum of squares F-test).

Although whole cell transmembrane currents in ten out of fourteen patch clamped SaOS-2 osteoblastic cells showed an apparent sensitivity to 100 µM ruthenium red (Figure 1, A-B), mean transmembrane currents (normalized for capacitance) were not significantly decreased by 100 µM ruthenium red either in 2 mM extracellular Ca^2+^ ([Ca^2+^]_O_; *P* = 0.21, n = 5; Figure 1, C) or in 0 mM [Ca^2+^]_O_ (*P* = 0.20, n = 5; data not shown). Under the experimental conditions, whole cell transmembrane currents showed no rectification and the relationship between current and voltage was best fitted to a straight line (Figure 1, C). In contrast, mean transmembrane currents (normalized for capacitance) in the human bronchial epithelial cell line 16HBE14o-, which we have previously shown to express TRPV6 [21] did display sensitivity to 100 µM ruthenium red (Figure 1, D, *P* = 0.00018), and the ruthenium red sensitive currents in these cells accounted for 31 ± 4% (mean ± SEM) of the total current at 60 mV holding potential.

### Assessment of TRPV ion channel mediated ^45^Ca uptake

**Figure 2:**
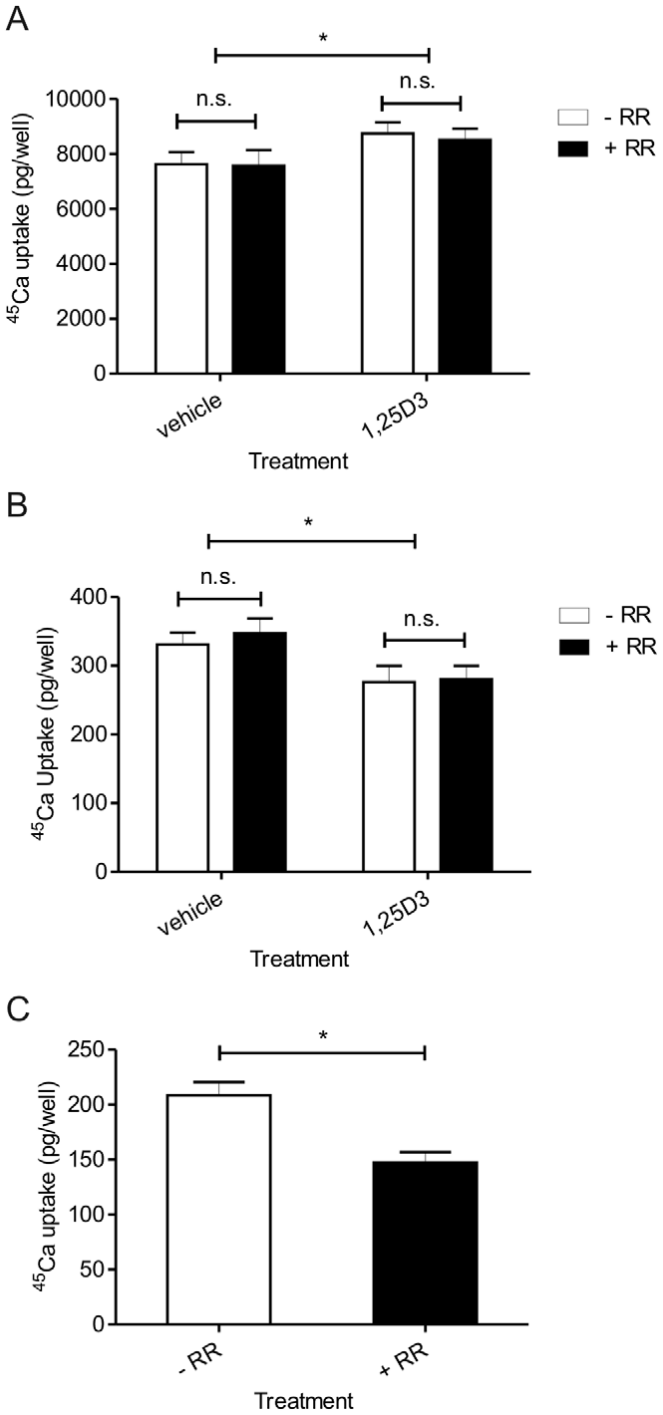
^45^Ca uptake assays. Uptake of ^45^Ca by SaOS-2 cells (A: *P* = 0.76) , TE-85 cells (B: *P* = 0.69) and 16HBE14o- cells (C: *P* = 0.0003) in the absence (open bar) and presence (solid bar) of 100 µM ruthenium red. Treatment of SaOS-2 cells with 1,25D3 (10 nM for 7 days) resulted in an increase in ^45^Ca uptake (*P* = 0.04) that was insensitive to ruthenium red. Treatment of TE-85 cells with 1,25D3 (24 h) resulted in a ruthenium red insensitive decrease in ^45^Ca uptake (*P* = 0.03). n = 3 experiments with 6 replicates per treatment for all cell lines. * *P*<0.05, n.s. not statistically significant.

Ruthenium red (100 µM) had no significant effect on uptake of ^45^Ca by SaOS-2 cells (*P* = 0.77) or TE-85 cells (*P* = 0.69; Figure 2), either in the absence or presence of 10 nM 1,25D3. Treatment with 1,25D3 for seven days resulted in a statistically significant increase in ^45^Ca uptake in SaOS-2 cells (*P* = 0.04) that was not sensitive to ruthenium red (Figure 2, A). A statistically significant, ruthenium red insensitive, decrease in ^45^Ca uptake was observed in TE-85 cells (*P* = 0.03) in response to 24 h treatment with 1,25D3 (Figure 2, B). In contrast, 16HBE14o- cells showed sensitivity to ruthenium red (Figure 2, C) and the ruthenium red sensitive uptake accounted for 27±5% of the total uptake.

### TRPV6 gene transcript expression in cells and tissues

**Figure 3:**
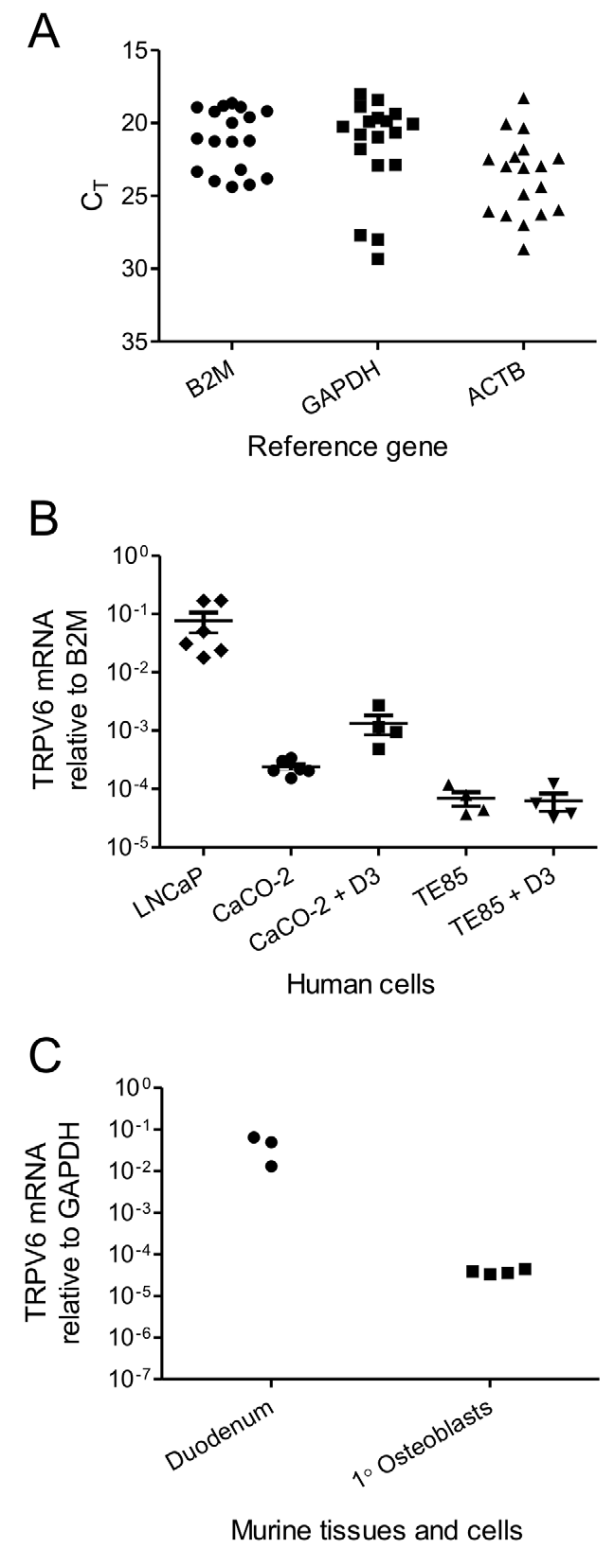
TRPV6 mRNA expression in human and murine cells and tissues. A: Variation in reference gene expression in human cDNA templates. B: TRPV6 mRNA expression in human cells, relative to B2M. D3: 12 h with 10 nM 1,25D3. Data are individual measures with mean ± SEM. C: TRPV6 mRNA expression in murine duodenum and in primary murine calvarial osteoblasts, relative to B2M (n = 3 for duodenum and n = 4 for calvarial osteoblasts). * *P*<0.05, n.s. not statistically significant.

We selected B2M as the reference for human TRPV6 mRNA expression as it was the least variable of three reference genes tested in the samples we analyzed (Figure 3, A). For murine TRPV6 mRNA expression, we selected GAPDH as the least variable reference (data not shown). TRPV6 mRNA was readily detected in LNCaP and CaCO-2 cells, which displayed the highest levels for the human cells we examined (7.7±3.0 × 10^−2^ and 2.4±0.3×10^−4^ relative to B2M; Figure 3, B). Treatment for 12 h with 1,25D3 (10 nM) resulted in a significant (*P* = 0.023) up regulation of TRPV6 mRNA in CaCO-2 cells by nearly 10-fold (Figure 3, B). In TE-85 cells, mRNA for TRPV6 was expressed near the limit of detection, at 6.9±1.9 × 10^−5^ relative to B2M. TRPV6 transcript levels and were not altered by treatment with 1,25D3 in these cells (*P* = 0.815; Figure 3, B). In SaOS-2 cells, TRPV6 mRNA expression was undetectable within 40 qPCR cycles, whether under basal conditions or following stimulation with 1,25D3 (data not shown). TRPV6 mRNA expression was not significantly altered (*P* = 0.842) in TE-85 cells grown in osteogenic medium for 7 days, and remained undetectable in SaOS-2 cells with this treatment (data not shown). TRPV6 mRNA was readily detected in murine duodenum at between 1.3 and 6.5 × 10^−2^ relative to GAPDH (Figure 3, C). In murine primary calvarial osteoblasts, TRPV6 mRNA was detected at 3.83±0.24×10^−5^ relative to GAPDH (Figure 3, C).

### Immunohistochemistry for TRPV6 in cells and tissues

**Figure 4:**
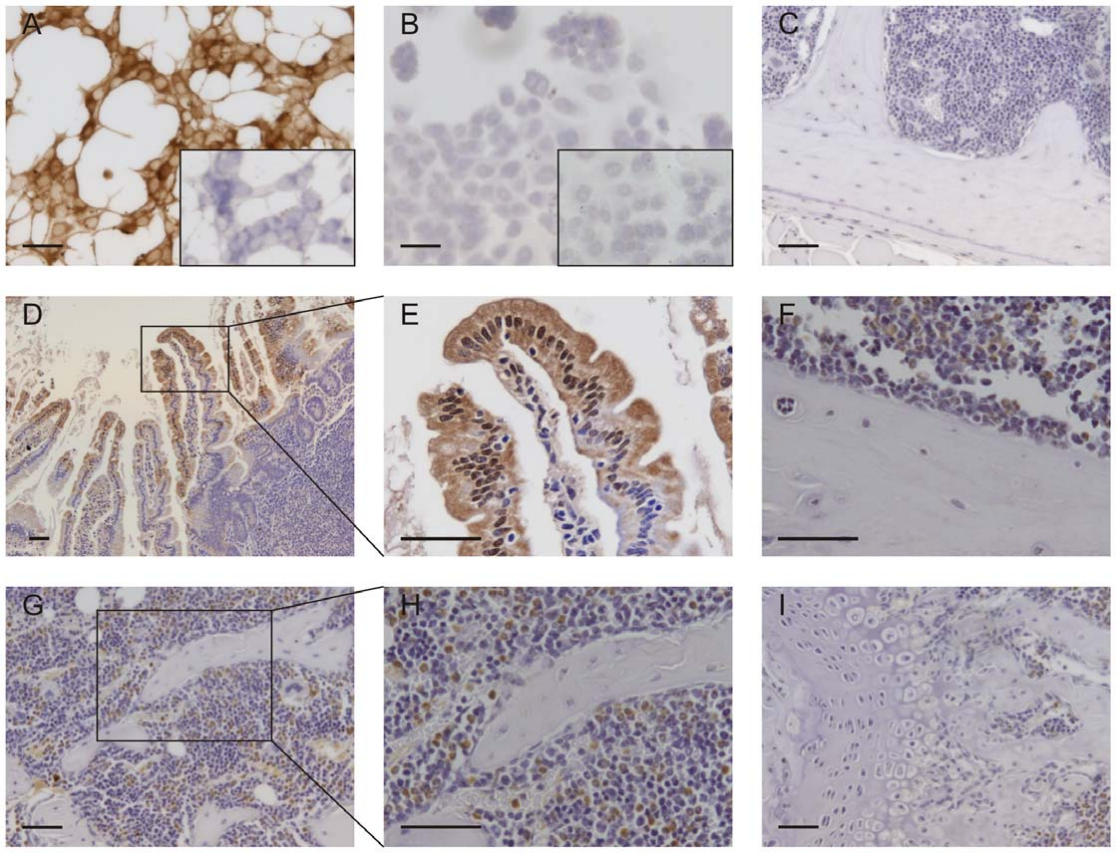
TRPV6 protein expression in bone cells and tissues. A, B: TRPV6 expression in LNCaP (A) and TE-85 (B) cells. An inset image shows staining with normal goat IgG (1 µg/mL) in each cell line. The slides in A and B were processed simultaneously. C: Staining with normal goat IgG (1 µg/mL) in mouse bone. D, E: TRPV6 expression in normal murine duodenum (positive control). F-I: TRPV6 expression in normal murine bone. Tissues from 6-week old male C57Bl/6J mice were fixed in 10% buffered formalin for 48 hours. Bones were decalcified for 4 weeks in EDTA/PFA. Images are representative of three mice. Slides in C-I were processed simultaneously. Scale bar: 50 µm in all panels.

TRPV6 expression, revealed by DAB immunoperoxidase staining, was considerably stronger in LNCaP cells (Figure 4, A) than in TE-85 cells (Figure 4, B) when cultured cells were processed simultaneously. In murine duodenum, staining for TRPV6 was strong in enterocyte cells of the intestinal villi (Figure 4, D&E) with a concentration of expression at the apical cell membrane (luminal facing) at the tips of the villi (Figure 4, E). In contrast, there was little or no staining for TRPV6 in osteoblasts either at the endocortical surface (Figure 4, F) or on trabecular bone (Figure 4, G&H). TRPV6 was not detected in cortical or trabecular osteocytes (Figure 3, F-H) nor in the growth plate (Figure 4, I). In contrast, strong staining was observed in a proportion of cells throughout the bone marrow, principally in cells with multilobular nuclei (Figure 4, F-I).

## Discussion

Our initial aim was to characterize the contribution of TRPV6 ion channels to Ca^2+^ currents in whole cell patch clamped SaOS-2 osteosarcoma cells. We selected SaOS-2 cells for our electrophysiological studies as they have been widely used as a model of differentiated osteoblast lineage cells that can form mineralized matrix both *in vitro* [22] and *in vivo* [23]. We used ruthenium red to differentiate TRPV6-mediated ion currents from other Ca^2+^ currents as it has been reported as an inhibitor of TRPV ion channels, with IC_50_ values of 9±1 µM and 121±13 nM for TRPV6 and TRPV5, respectively [19]. We are aware of the potential inhibition by ruthenium red of other ion channels, especially since it virtually abolishes ryanodine receptor mediated Ca^2+^ currents in rat and rabbit osteoclasts at similar concentrations to what we have used here [24]. However, we did not detect any ruthenium red sensitive transmembrane currents in SaOS-2 cells. In contrast, averaged and normalized whole cell currents in 16HBE14o- cells, which we have previously shown to express TRPV6 [21], did show sensitivity to ruthenium red. However, in the 16HBE14o- cell line the reversal potential was close to 0 mV and was not characteristic of TRPV6 Ca^2+^ ion channels observed in overexpression systems such as Chinese hamster ovary (CHO) cells [25], for which a value of 49±5 mV has been reported. To date, there are no publications describing the electrophysiological characteristics of native TRPV6 ion channels in any cell type and therefore the difference in the reversal potential from our 16HBE14o- cell data and that from CHO cells remains unclear. Further studies are needed on the requirement and role of accessory molecules which may modulate the behavior and electrophysiological characteristics of TRPV6 ion channels in their native configuration. In both SaOS-2 and 16HBE14o- cells, the magnitude of the currents and the linearity of the current-voltage relationship could suggest leak current arising from poor patch sealing but the time-dependent inactivation of channels evident in our traces argues against this (see Figure 1 A, +100 mV trace). Few studies have characterized osteoblastic cells by whole cell patch clamping. In the only published study using whole cell patch clamping in SaOS-2 cells, Laketic-Ljubojevic *et al*. [26] demonstrated the presence of functional N-methyl D-aspartate receptors. Whole cell and excised patch voltage-clamp studies in a variety of other osteoblastic and osteogenic cells have demonstrated a variety of ion conductances, including a transient outward rectifying K^+^ conduction, a calcium activated K^+^ conductance, chloride and phosphate mediated conductances, and stretch activated cation channels [27], [28]. Our pipette solution contained Cs^+^ to block K^+^ ion channels and we therefore expect that the main conductance in SaOS-2 cells was mediated by non-selective cation and chloride channels. Our laboratory has previously shown that the association of TRPV6 with annexin-2/S100A10 and the formation of annexin-2/S100A10 complex in airway and gut epithelia are both dependent on cyclic 3′,5′-adenosine monophosphate (cAMP) and cAMP-dependent protein kinase (PKA, EC 2.7.1.37) [21], and that calcium influx in Caco-2 cells is attenuated by PKA and calcineurin A inhibition [21]. We therefore included forskolin and IBMX in our bath solution to elevate cAMP and maximise the opportunity for observing TRPV6-mediated currents. Increasing cAMP may also have affected other cAMP-dependent ion channels, which might account for the difference in magnitude of native currents between our studies and that previously reported in SaOS-2 cells by Laketic-Ljubojevic *et al*. [26].

We did not observe a significant effect of ruthenium red on ^45^Ca uptake in either SaOS-2 or TE-85 cells. In contrast, uptake in TRPV6-expressing 16HBE14o- cells was significantly inhibited by ruthenium red, accounting for approximately 27% of the total uptake. While Ca^2+^ uptake in both SaOS-2 and TE85 cells was insensitive to ruthenium red, we observed significant ruthenium red independent changes in uptake of Ca^2+^ in these cells in response to treatment with 1,25D3, suggesting that 1,25D3 modulates the expression or activity of calcium selective ion channels other than TRPV6 in these cells. We did not assess uptake in osteoblastic cells under more differentiated conditions because it is difficult to distinguish active cellular calcium uptake from the binding of calcium to extracellular proteins and mineral that are generated over time in these cultures.

The results from our two functional assays for TRPV6 are consistent with the conclusions of the recent study by van der Eerden *et al*. [20] who overexpressed TRPV6 in a simian virus-immortalized human fetal osteoblast-like (SV-HFO) cell line and showed that it had no effect on mineralization *in vitro*. The lack of ruthenium red sensitivity in our assays led us to question whether TRPV6 was expressed in the osteoblastic cells used in our studies. Using a qPCR assay, we observed that TRPV6 transcripts were below the assay's limit of detection in SaOS-2 cells. TRPV6 mRNA expression was also very low in the human osteoblastic cell line TE85 and in primary murine calvarial osteoblasts, contrasting with earlier reports in the literature of TRPV6 expression in human bone [18] and murine osteoblasts [17]. The levels of TRPV6 transcripts measured in our assays also contrast strongly with the recent data from van der Eerden *et al*. [20] who report TRPV6 transcripts at equal or higher levels to GAPDH in murine osteoblasts, in SV-HFO cells and in samples of human and murine femoral bone from which the bone marrow was removed. In our qPCR assays, GAPDH and B2M were expressed at levels within a factor of 10-fold of each other in most templates. Our values for TRPV6 expression are between 10^−4^ and 10^−5^ the level of B2M or GAPDH in human and murine osteoblasts respectively, which equate to between 10^5^- and 10^6^-fold lower than those reported in osteoblasts by van der Eerden *et al.* [20]. The same laboratory has reported TRPV6 mRNA levels in murine bone at around 50% of the level in murine duodenum [18], while we detected TRPV6 in murine osteoblasts at about 0.1% of the level in murine duodenum. This contrast is surprising since we have used identical primers and probe for TRPV6 in our qPCR assays [18], [20]. Our data for TRPV6 expression is consistent with the findings of Abed *et al*. [29], who did not detect TRPV6 in a variety of human and murine osteoblastic cells, but showed that other TRPV ion channels are present. Our data for TRPV6 mRNA levels in cells and tissue used as positive controls, i.e. LNCaP and CaCO-2 cells or murine duodenum, is consistent with other publications [4], [30], [31], and our data from primary murine calvarial osteoblasts relative to murine duodenum is consistent with data from Lieben *et al.* [31].

Despite the differences that we report in the level of gene expression, neither we nor van der Eerden *et al.* [20] could demonstrate an up regulation of TRPV6 in osteoblasts by 1,25D3. In our experiments, short-term treatment of SaOS-2 and TE85 cells with 1,25D3 for 12 hours had no effect on TRPV6 mRNA expression, while a similar treatment up-regulated TRPV6 expression in CaCO-2 cells. Seven days of treatment with an osteogenic medium containing ascorbate, dexamethasone and β-glycerophosphate also had no significant effect on TRPV6 expression either in SaOS-2 or TE85 cells. In their study, van der Eerden *et al.* [20] also showed that 1,25D3 did not regulate TRPV6 expression in either mineralizing or non-mineralizing cultures of human osteoblast-like cells.

In murine bone tissue, our immunohistochemical method showed that TRPV6 is principally expressed in bone marrow cells with multilobular nuclei suggestive of a myleocytic/leukocytic lineage. We detected little or no expression of TRPV6 in osteoblasts, osteocytes or growth plate chondrocytes in sections of murine bone. The high level of expression in bone marrow cells could possibly account for TRPV6 being detected in bone samples from which the marrow has largely been removed [20], but which inevitably will retain some traces of marrow cells. In sections of murine duodenum, processed as a positive control under identical conditions as and simultaneously with the bone samples, the distribution of staining for TRPV6 was consistent with its role in intestinal calcium uptake, with preferential staining of cells towards the tips of the intestinal villi and with a distinct distribution on apical cell membranes. Our findings in murine bone again contrast with the recent report of van der Eerden *et al.* [20] who have described “apical” TRPV6 expression in osteoblasts within a section of human (osteoarthritic) trabecular bone using an immunofluorescence method. Although we have used different antibodies and methods of immunodetection, on Western blots both antibodies appear to detect TRPV6 as two distinct bands [18], [21], that correspond to the core and glycosylated forms [18]. While our method using enzyme histochemical staining to reveal TRPV6 may not be particularly sensitive, our protocol allowed visualization of TRPV6 expression throughout the whole environment of bone, under conditions where its expression in murine duodenum was readily detected. Our data demonstrate that osteoblastic expression of TRPV6 is very low in relation to the level seen in the bone marrow myelocytic cells. The use of confocal microscopy combined with a secondary antibody labeled with Qdots® in the report by van der Eerden *et al.* [20] would provide very high sensitivity for detection of TRPV6 in their human bone sample, but it is difficult to relate their observed TRPV6 expression in osteoblasts with that in other cells in their bone sample as it would appear that the bone marrow may have been removed from their specimen and there is therefore little cellular or tissue context in their figure.

Although the level of expression of TRPV6 in osteoblasts is questioned here, there is no clear evidence either from our functional assays or from other laboratories that TRPV6 acts as a Ca^2+^ selective ion channel in osteoblastic cells. Our electrophysiological and calcium uptake assays show that TRPV6 contributes little to calcium handling in osteoblasts. While the initial phenotypic description of *Trpv6^-/-^* mice [11] suggested a potential role for TRPV6 within bone, a more recent and extensive study [31] has subsequently shown that bone mass is normal in both young and ageing *Trpv6* ^-/-^ mice receiving a normal (1%) calcium diet. In mice with a restricted (∼0.02%) calcium diet from weaning, the changes in bone formation and mineralization occurred equally in *Trpv6* ^-/-^ and wild-type mice, as an indirect consequence of the limited calcium supply [31]. Our data supports the conclusion that TRPV6 plays little physiological role in osteoblastic cells, and that the principal functions of TRPV6 in calcium homeostasis are to mediate active calcium uptake and reuptake in intestinal and renal epithelial tissues [31].

## Materials and Methods

### Materials

Gibco brand cell culture media, supplements and trypsin-EDTA solution were purchased from Fisher Scientific, UK. Recombinant human macrophage colony stimulating factor (MCSF) and recombinant murine receptor activator of nuclear factor κ-B ligand (RANKL) were purchased from Peprotech EU. Stock solutions of these cytokines were prepared at 5 µg/mL in sterile phosphate buffered saline (1x PBS; Gibco, UK) containing 0.1% (v/v) bovine serum albumin (BSA; Sigma, UK). RNA Superscript III reverse transcriptase and oligo-dT_20_ were purchased from Fisher Scientific. Universal qPCR Mastermix was purchased from Applied Biosystems (UK). For immunohistological studies a goat polyclonal IgG against TRPV6 (sc-31445) was purchased from Santa Cruz Biotechnology, USA, and a horseradish peroxidase-conjugated rabbit anti-goat IgG (A5420) was purchased from Sigma-Aldrich, UK. Rabbit serum, normal goat IgG and 3,3′-diaminobenzidine (DAB) peroxidase substrate solution were purchased from Vector Laboratories, Burlingame, USA and DePeX Mounting Medium purchased from VWR International, UK. ^45^CaCl_2_ was purchased from Amersham, UK. All other reagents were purchased from Sigma-Aldrich, UK unless otherwise stated.

### Cell lines

The human bronchial epithelial cell line 16HBE14o- was obtained from Dr. DC Gruenert, California Pacific Medical Center Research Institute, San Francisco, CA, USA. All other cell lines originated from the American Type Culture Collection and were purchased through LGC (UK). The human osteosarcoma-derived HOS/TE-85 osteoblastic cell line, the prostate carcinoma-derived LNCaP and the colon carcinoma-derived CaCO-2 cell lines were grown in Dulbecco's Modified Eagle's Medium (DMEM) containing Glutamax®, supplemented with 10% FCS (Biowhittaker, Maryland, USA), 100U/mL penicillin and 100 µg/mL streptomycin. CaCO-2 cells were additionally supplemented with 1% (v/v) 100x non-essential amino acids and 0.06% (v/v) gentamicin. The human osteosarcoma-derived SaOS-2 osteoblastic cell line was grown in α-MEM supplemented as for TE-85 cells. 16HBE14o- cells were grown in medium 199 containing 10% fetal calf serum, penicillin (100 U/ml) streptomycin (0.1 mg/ml) and l-glutamine (2 mM) as described previously [21]. Media preparations as described here are referred to as complete growth medium below. Cells were maintained at 37C in tissue culture grade flasks or multiwell dishes (Corning Incorporated, USA) in a humidified atmosphere of 95% air and 5% CO_2_.

### Electrophysiology

SaOS-2 and 16HBE14o- cells were plated at low density on 13 mm plastic coverslips (Agar Scientific, UK) in a 10 cm tissue culture dish (Iwaki, Ashai Glass Co., Japan). Whole-cell patch clamp experiments [32] were performed in a Perspex bath clasped onto the stage of an Olympus IX70 inverted microscope. Thirty minutes prior to patching, a coverslip was transferred to the Perspex bath which contained an extracellular solution consisting of 120 mM CsCl, 10 mM HEPES titrated to pH 7.4 with CsOH, 10 µM forskolin and 100 µM 3-isobutyl-1-methylxanthine (IBMX). The pipette solution consisted of 20 mM CsCl, 100 mM Cs aspartate, 10 mM HEPES titrated to pH 7.4 with CsOH, 1 mM MgCl_2_, 10 mM 1,2-bis(o-aminophenoxy)ethane-N,N,N',N'-tetra acetic acid and 4 mM Na_2_ATP in dH_2_O, and either 0 mM (low) or 2 mM (high) CaCl_2_. Potentials were driven by v6 pCLAMP software with a Digidata 1200 Series interface (Axon Instruments, UK) for membrane voltage steps between −100 mV and +100 mV from a holding potential of −40 mV. High frequency electrical noise was reduced by passing signals through an 8-pole Low Pass filter at 5 KHz, and by placing a shield in front of the microscope. On achieving whole-cell configuration, cells were perfused under gravity with extracellular solution and current at steady state was recorded with a L/M EPC7 amplifier (Heka Electronik, Germany) with recordings stored directly to the computer hard drive. Capacitance of each patched cell at steady state was also recorded. The contribution of TRPV6 channel activity to whole cell current was assessed by perfusing cells with extracellular solution supplemented with ruthenium red (100 µM) [19], [33]. Ca^2+^ selectivity of whole-cell currents was assessed by perfusing cells with extracellular solution containing 5 mM mannitol in place of 2 mM CaCl_2_. The ruthenium red-sensitive current was calculated as the whole-cell current in the presence of ruthenium red subtracted from the current obtained in the absence of ruthenium red at each potential in both extracellular solutions.

### Calcium uptake assays

For ^45^Ca uptake assays, SaOS-2 TE-85 and 16HBE14o- cells were plated in 12-well multi-well plates at 2×10^5^ cells per well. SaOS-2 cells were incubated for 2 days in complete growth medium and thereafter supplemented with dexamethasone (10 nM), β-glycerophosphate (10 mM) and ascorbic acid (500 ng/mL) to promote osteoblastic differentiation over 7 days [34]. During the final 3 days the medium was supplemented with 1,25D3 (10 nM) or with vehicle (ethanol, 0.1% v/v). TE-85 cells and 16HBE14o- cells were incubated for 2 days in complete growth medium. Subsequently for all preparations, the growth medium was replaced with buffered saline solution (140 mM NaCl, 10 mM HEPES titrated to pH 7.4 with NaOH, 2 mM CaCl_2_, 1 mM MgCl_2_,5 mM KCl, 10 µM verapamil and 10 µM felodopine) for 10 minutes and ^45^Ca uptake was assayed as described previously [21]. Briefly, cells were incubated for 30 min at 37C in 5% CO_2_ in buffer solution supplemented with forskolin (10 µM) and IBMX (100 µM). During the last minute, cells were exposed to ^45^CaCl_2_ (5 µCi/mg), washed three times in 30 seconds with ice-cold buffered saline then lysed using 10% (w/v) sodium dodecyl sulfate in buffered saline over 2 hours. ^45^Ca levels were measured by Direct DPM scintillation counting (Packard Tri-Carb 1600 TR). Assay treatments were performed in sextuplicate and all assays were repeated three times. Ca^2+^ uptake was quantified as picogram ^45^Ca per well. TRPV ion channel-mediated uptake was calculated as the difference between uptake in the presence and absence of ruthenium red (100 µM).

### Gene expression assays

For analysis of gene expression, the osteoblastic cell lines SaOS-2 and TE-85 were incubated with 1,25D3 (10 nM) in molecular grade ethanol or with ethanol alone (0.1% v/v, vehicle control) for 3 days. Primary murine calvarial osteoblasts were prepared as previously described [35] from 2-day old C57BL/6 mice. For each preparation, recovered cells were grown to confluence in two T75 tissue culture flasks in α-MEM complete growth medium. Total RNA was extracted from cultured cells using the GeneElute™ Mammalian Total RNA MiniPrep Kit (Sigma-Aldrich) immediately after discarding culture medium. Total RNA was extracted from mouse tissues using Tri-reagent (Sigma, 1 mL/100 mg tissue). All RNA extracts were treated with DNase 1 (Sigma-Aldrich, 1 unit/µL) for 15 min at room temperature. Following DNase treatment, RNA was quantified and checked for purity using a NanoDrop ND-1000 Spectrophotometer (NanoDrop Technologies Incorporated, Wilmington, USA). All samples had an OD_260_/OD_280_ ratio of between 1.90 and 2.04. For all samples, 1 µg of total RNA was reverse transcribed using SuperScript III reverse transcriptase (10 U/µL) and oligo-dT_20_ primer (2.5 µM) in a total volume of 20 µL. 1 µL of each cDNA sample was used in 10 µL qPCR assays to measure expression of TRPV6 and the reference genes *B2M*/*B2m*, *GAPDH/Gapdh* and *ACTB/ActB* using On Demand TaqMan™ Gene Expression Assays or previously published primer/probe sets [18] as indicated in Table 1. Assays were performed in 384-well microplates (Grenier bio-one, UK) using a 7900HT Fast Real-Time PCR system (Applied Biosystems, UK). For each template, TRPV6 and the appropriate reference genes were assayed on the same plate in three assay replicates. For human cell line templates, cDNA from LNCaP and CaCO-2 cells were included as positive controls on the same plates. cDNA from mouse duodenum was used as a positive control for TRPV6 gene transcript expression in murine bone cells. Negative controls of nuclease-free water and RT- templates were also included in all assay runs. TRPV6 gene transcript expression was calculated relative to reference genes based on the *ddC_T_* method: *ddC_T_  =  2^–dCT^*, where *dC_T  = _ C_T_ (gene of interest) – C_T_ (reference gene)* [36].

**Table 1:** Primers for qPCR.

Target gene	Primer/probe sequence or Assay ID	
*Trpv6* 1	Forward:	5′-ATCCGCCGCTATGCACA-3′
	Reverse:	5′-AGTTTTTCTCCTGAATCTTTTTCCA-3′
	Probe:	5′-TTCCAGCAACAAGATGGCCTCTACTCTGA-3′
*Gapdh*	Mm99999915_g1^2^	
*ActB*	Mm00607939_s1^2^	
*TRPV6*	Hs00367960_m1^2^	
*GAPDH*	Hs99999905_m1^2^	
*ACTB*	Hs99999903_m1^2^	
*B2M*	Hs99999907_m1^2^	

1. Published by Nijenhuis *et al.*, 2003 [18].

2. On Demand TaqMan™ Gene Expression Assays (Applied Biosystems, Life Technologies Corporation).

### Immunohistochemistry

Tissues were obtained from animals killed under a UK Home Office Schedule 1 procedure and did not require local ethical committee approval. Duodenum and femurs from 6 week old male C57Bl/6J mice were trimmed of excess muscle and fixed in 10% buffered formalin (pH 7.0) for 48 hours. Bones were subsequently decalcified in buffered 15% EDTA/0.5% paraformaldehyde (pH 8.0) solution at room temperature and maintained on a slowly oscillating platform for 4 weeks with weekly changes of solution. Tissues were embedded in paraffin wax immediately following fixation (duodenum) or decalcification (femurs). Tissues were sectioned (3 µm) and mounted onto glass slides then processed simultaneously. Sections were dewaxed in xylene followed by 99% ethanol (2x 5 min each), then immersed in ice-cold methanol for 10 minutes. Slides were treated with 1% H_2_O_2_ (v/v) in methanol for 30 minutes then washed in three changes of dH_2_O. TE-85 cells and LNCaP cells grown on glass coverslips were fixed in ice-cold methanol for 10 minutes. Slides with tissues and coverslips with cells were blocked using 5% (v/v) rabbit serum in 1x PBS (blocking solution) for 30 minutes, then incubated for 16 hours at 4C with either a goat polyclonal IgG against TRPV6 (1∶200) or normal goat IgG (1 µg/mL) diluted in blocking solution. Samples were washed in PBS then incubated in blocking solution supplemented with horseradish peroxidase-conjugated rabbit anti-goat IgG (1∶200) for 40 minutes then washed in PBS. Samples were incubated in DAB peroxidase substrate solution for 10 minutes and washed in dH_2_O before counterstaining with hematoxylin for 30 seconds. Slides and coverslips were dehydrated in graded ethanol solutions (70%, 95%, 2x 99%) and xylene (5 min each), then mounted using DePeX. Images were captured using a Leica DMI4000B microscope equipped with a Leica DFC300FX digital camera and LAS image capture software.

### Statistical analysis

All results are expressed as means ± standard error (SEM). For electrophysiological data, currents corrected for capacitance across the applied voltage range were fitted to the best fit line, which was either linear or a 2^nd^ order polynomial (quadratic) function for these data. Differences in current profiles between experimental conditions were determined by comparing best fit models using the extra sum of squares F-test in GraphPad Prism (La Jolla). Other comparisons were undertaken using ANOVA, t-tests or the Mann-Whitney U-test. Statistical significance was assumed at the 5% level.
